# Is Mean Platelet Volume Really a Severity Marker for Obstructive Sleep Apnea Syndrome without Comorbidities?

**DOI:** 10.1155/2014/754839

**Published:** 2014-09-17

**Authors:** Sinem Nedime Sökücü, Cengiz Özdemir, Levent Dalar, Levent Karasulu, Şenay Aydın, Sedat Altın

**Affiliations:** ^1^Sleep Laboratory, Yedikule Chest Disease and Thoracic Surgery Training and Research Hospital, Zeytinburnu, Istanbul, Turkey; ^2^Department of Pulmonary Medicine, School of Medicine, Istanbul Bilim University, Sisli, 34600 Istanbul, Turkey

## Abstract

Obstructive sleep apnea syndrome (OSAS) is a common disorder that can lead to significant cardiovascular complications. Several studies have reported increased platelet activation and aggregation in patients with OSAS. In this study we aimed to show a correlation between mean platelet volume (MPV) and severity of OSAS in patients with OSAS without any overt cardiac disease or diabetes. The polysomnography recordings of 556 consecutive patients admitted to the sleep laboratory between January 2012 and July 2012 were retrospectively evaluated. The relationship between polysomnographic parameters and biochemical parameters was assessed. Polysomnographic results of 200 patients (154 males [77%]; mean age, 44.5 ± 11.4 years) were included. No correlation was observed between MPV and the average oxygen saturation index, the minimum desaturation index, or the oxygen desaturation index in the study population as well as in severe OSAS group (AHI > 30). The only correlation was found between MPV and AHI in the severe OSAS group (*P* = 0.010). MPV was not correlated with OSAS severity in patients without any overt cardiac disease or diabetes. These findings raise doubts about the suggestion that MPV might be a marker for OSAS severity, as recommended in earlier studies. Thus, further prospective data are needed.

## 1. Introduction

Obstructive sleep apnea (OSA) is characterized by recurrent episodes of partial or complete upper airway obstruction during sleep. It occurs as combined episodes of apnea and hypopnea that cause sleep fragmentation or excessive daytime sleepiness. It is a common disorder affecting 2% and 4% of middle aged women and men, respectively [1]. The severity of OSAS is estimated by the number of apnea-hypopnea episodes per hour of sleep and is expressed as the apnea-hypopnea index (AHI) [2]. Although the underlying mechanisms and etiologies are not completely understood, OSAS can lead to significant cardiovascular complications [3], including heart failure, acute myocardial infarction, arrhythmias, hypertension, pulmonary hypertension, and stroke [4]. Increased platelet activation and aggregation are closely related to cardiovascular complications [5]. Several studies have reported increased platelet activation and aggregation in patients with OSAS [6, 7].

It has been shown that platelet size, measured by mean platelet volume (MPV), correlates with platelet reactivity and is an easy and useful tool for indirect monitoring of platelet activity. Larger platelets have higher thrombotic potential [8]. MPV plays an important role in the pathophysiology of cardiovascular diseases [8, 9]. The association between increased platelet activation and aggregation is closely associated with cardiovascular complications. Increased MPV occurs in patients with hypertension, hypercholesterolemia, diabetes mellitus, acute myocardial infarction, acute ischemic stroke, and coronary artery calcification [10, 11].

The relationship between MPV and disease severity in patients with OSAS has been evaluated and MPV increases in patients with OSAS when used as an indicator of platelet activation [12, 13]. Continuous positive airway pressure (CPAP) therapy is shown to decrease platelet activation in patients with OSAS [14, 15]. In this study, relation between MPV and severity of OSAS in nondiabetic patients without any overt cardiac disease was evaluated.

## 2. Patients and Methods

### 2.1. Patients

A total of 556 patients consecutively admitted to the sleep laboratory between January 2012 and July 2012 who are with symptoms of nocturnal snoring and/or excess daytime sleepiness and who underwent a polysomnographic evaluation were retrospectively evaluated. Patients having AHİ < 5 were grouped as control and others were grouped according to their AHI value as mild, moderate, and severe OSAS.

Inclusion criteria were patients who are with symptoms of nocturnal snoring and/or excessive daytime sleepiness and who underwent polysomnographic evaluation at our sleep laboratory. Exclusion criteria were any known cardiac disease (congestive heart failure, ischemic vascular disease, or arrhythmias), lung disease (chronic obstructive pulmonary disease and asthma), diabetes mellitus (defined as fasting plasma glucose >126 mg/dL and/or antidiabetic treatment), chronic renal or hepatic diseases, use of acetylsalicylic acid or any other antiaggregant therapy (dipyridamole, ticlopidine, and clopidogrel) within the last month, abnormal haematocrit and/or abnormal white blood cell count and/or abnormal platelet number, and pure or mainly central apnea on a polysomnographic evaluation. The cardiac disease history evaluation was conducted through a detailed medical history and an evaluation of earlier ECGs, tests, and coronary angiography. Use of acetylsalicylic acid or other antiaggregant therapy was evaluated not only by history taking but also by assessing 3 months of pharmacy records. Rapid eye movement (REM) induced OSAS and positional OSAS patients were excluded from the study because in those patient groups severity of the disease changes day to day depending on the REM percentage of the sleep and time duration spent in supine position. For our study the definition of positional OSAS is accepted as total AHI > 5, nonsupine AHI < 5, and supine AHI/nonsupine AHI ≥ 2. The definition of REM-OSAS is accepted as total AHI > 5, REM AHI < 5, and REM AHI/non-REM AHI ≥ 2.

**Figure 1:**
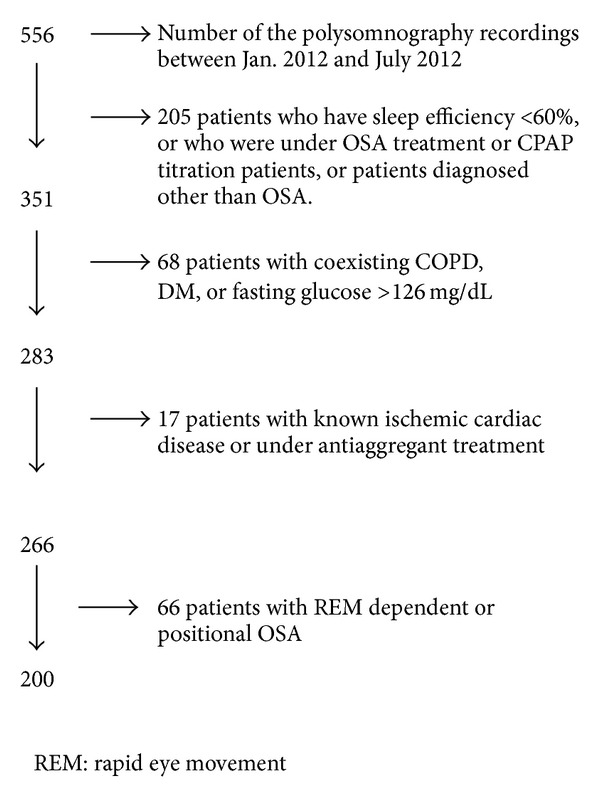
Exclusion chart of patients.

In total, 200 of the 556 met the inclusion and exclusion criteria and were enrolled in the study (Figure 1). Detailed medical history, physical examination, electrocardiogram (ECG), and chest X-ray were assessed from the patient folders. This retrospective study protocol was approved by the institutional ethics committee. Informed written consent was obtained from all subjects before the polysomnography.

### 2.2. Procedure

Standard overnight polysomnography was performed in all patients using an Embla N7000 (Embla, Medcare Flaga, Iceland) data acquisition and analysis system in the sleep laboratory from 22.00 to 06.00 h. The physiological signals monitored included EEG (C4-M1, C3-M2, O2-M1, and O1-M2), electrooculography, and submental EMG. The following were also measured: ribcage and abdominal effort measured by respiratory inductive plethysmography (RIP) (XactTrace, Medcare Flaga), body position, measured by calibrated sensor, snoring sound measured with a piezoelectric sensor, and oronasal flow measured with a nasal pressure cannula (Medcare Flaga), SpO_2_ (8000J, Nonin Medical, Plymouth, MN, USA) with averaging time set at 3 seconds. The ECG (lead II) was sampled at 512 Hz. Sleep stages and arousals were scored using the Somnologica Studio software package (Medcare Flaga) according to standard criteria [16] by two experienced scorers who had 80–95% concordance with each other. Respiratory events were scored as follows. Apnea was defined as a cessation of airflow for ≥10 seconds. Apnea was classified as obstructive in the presence of continued movement in the RIP and as central in the absence of movement in the RIP. Hypopnea was defined as a ≥50% reduction in oronasal flow amplitude ≥10 seconds, accompanied by ≥3% desaturation or arousal. Classification of a hypopnea as obstructive, central, or mixed performed calibrated respiratory inductance plethysmography. Hypopnea was classified as obstructive in the presence of continued movement in the RIP [16]. The oxygen desaturation index (ODI) is the number of times per hour of sleep in which the blood's oxygen level drops by 3 percent or more from baseline.

### 2.3. Blood Assays

Fasting (8 hours) venous blood samples were drawn from the antecubital vein between 7 and 8 AM after polysomnography and after a 20 min rest. Tripotassium ethylenediaminetetraacetic acid based anticoagulated blood samples were drawn and assessed within 30 minutes. Complete blood count analyses were performed using the Abbott Cell-Dyne 3700 System (Abbott Diagnostics, Santa Clara, CA, USA) and biochemical analyses were performed using the Olympus AU2700 Plus Analyzer (Beckman Coulter, Tokyo, Japan).

### 2.4. Statistical Analysis

The statistical analysis was performed with SPSS for Windows version 16.0 (SPSS, Chicago, IL, USA). All variables were tested for normality with the Kolmogorov-Smirnov test. Normally distributed continuous variables are expressed as mean ± standard deviation. Non-normally distributed continuous variables are summarized as medians.

Categorical variables are expressed as numbers (percentages). Comparisons between independent groups were made using the Mann-Whitney *U*  test. Correlations between noncontinuous variables and continuous variables with a nonnormal distribution were assessed using Spearman's correlation. Correlations between continuous variables were assessed using Pearson's correlation. Comparisons between groups were evaluated by one-way analysis of variance followed by the Bonferroni method. A *P* < 0.05 was considered statistically significant.

## 3. Results

A total of 200 patients were included (154 males [77%]; mean age, 44.5 ± 11.4 years). The study subjects were categorized into four groups according to AHI (<5, normal; 5–15, mild OSAS; 15–30, moderate OSAS; >30, severe OSAS). As the severity of OSAS increased, male, older, heavier, and smoker patients became predominant (Table 1). A significant difference was found between cases with AHI < 5 and those with severe OSAS (AHI > 30) in terms of sex, age, body mass index (BMI), smoking ratio, and pack-year smoking history (*P* = 0.001, *P* = 0.014, *P* = 0.001, *P* = 0.231, and *P* = 0.015, resp.). No difference was found in terms of hypertensive patient ratios between the groups.

The polysomnographic characteristics of the groups are shown in Table 2. No differences were observed between the controls and patients with mild, moderate, or severe OSAS according to white blood cells, red blood cells, haemoglobin, haematocrit, platelets, MPV, platelet distribution width (PDW), glucose, creatinine, total cholesterol, or low-density lipoprotein cholesterol (Table 3). Both high-density lipoprotein (HDL) and triglycerides were significantly higher in the severe OSAS group, as compared to those in the normal population (*P* = 0.001 and *P* = 0.013).

**Figure 2:**
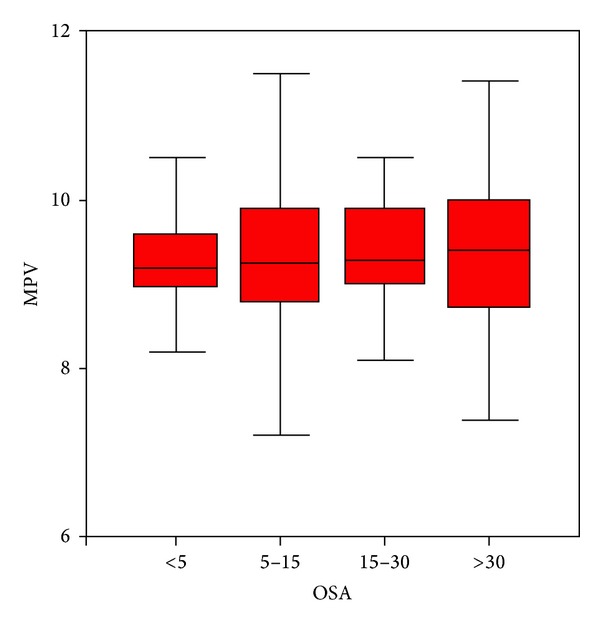
Box plot graphic of MPV and OSAS groups.

No correlation was observed between MPV and AHI, average saturation, minimum desaturation, time duration with SpO_2_ < 90%, oxygen desaturation index, or OSAS groups in the whole patient population (Table 4) (Figure 2). Also no correlation was found between MPV and average saturation, minimum desaturation, or the oxygen desaturation index in the severe OSAS subgroup. A correlation was found between MPV and AHI in the severe OSAS group (*P* = 0.010) (Table 5). No correlation was found between MPV and smoking history (*P* = 0.240) and sex (*P* = 0.887).

## 4. Discussion

The only correlation that was found in our study is that MPV is positively correlated with AHI in severe OSAS group but no significant relationship between OSAS and hypoxia parameters and MPV or PDW was found. Results of earlier studies conducted in nondiabetic subjects with OSAS showed that MPV and PDW are associated with the degree of hypoxia and OSAS severity. These results underline the importance of OSAS as a risk factor for vascular atherothrombotic disease and MPV as a risk factor [12, 13]. However, the relationship between OSAS severity and MPV could disappear if a more detailed history was taken, and all patients with cardiac and lung diseases were excluded.

MPV reflects platelet activity. The exact mechanism of platelet activation in patients with OSAS is unclear but possible mechanisms are an indirect effect of increased sympathetic activation causing catecholamine discharge that activates platelets, chronic intermittent hypoxia causing platelet activation directly, and chronic inflammation [17, 18]. In a previous study, a significant difference was found between the control group and severe OSAS in terms of MPV values, and severe OSAS was independently correlated with AHI and the desaturation index in a multivariate regression analysis. We found no correlation between any group in terms of MPV, AHI, or the desaturation index [12]. We only found a correlation between MPV and AHI in patients with severe OSAS. Also an explanation for this could be that in our control and mild OSAS groups ODI and time duration with Sat 02 <90% were relatively less compared to severe OSAS group. In a recently published study, MPV was not found to be different in mild and moderate OSAS but it was significantly high in severe OSAS. Also as reported by Karakas et al. in our study desaturation ratios were found to be similar in mild and moderate OSAS. [19]. This could be because of the fact that as the severity of the disease increases, patients become more prone to cardiovascular risks so we did not observe this relation in mild and moderate OSAS groups. As the disease severity increases, systemic inflammation also increases and this will affect MPV value just like other parameters of systemic inflammation. As the disease gets severer systemic reflections may increase and this could point to the relation between cytokine levels generating systemic inflammation and MPV.

Platelet count and MPV are modified by various biosocial and lifestyle factors such as race, age, gender, smoking, alcohol consumption, and physical activity [20–22]. An earlier study found no significant difference between controls and the severe OSAS group in terms of age, male ratio, and smoking habits, unlike our study, but there was a significant difference in terms of BMI, as in our study [12]. However, in the study by Nena et al., not all of these properties were mentioned in the groups [13]. Patients in our severe OSAS group were predominantly male and older, had more pack-year smoking history, and were more obese, as compared to controls, and all four criteria could affect MPV in a positive way. However, we did not find any differences in these variables in our patient population, as compared to controls.

Smoking increases MPV in older patients with risk factors for atherosclerosis. In a study conducted by Kario et al., increased MPV in smoking patients decreased after the patients stopped smoking [23]. This effect could not be demonstrated in younger smoking patients [24]. Based on these results, given that our control group was younger with less smoking and our severe OSAS group was older with more smoking, we should have seen an increased MPV in the severe OSAS group; however, there were no significant differences between the two groups in terms of MPV.

The relationship between MPV and gender has been studied in different populations. The direction and magnitude of this association may differ according to gender as shown in patients with metabolic syndrome (MS). Platelet counts in women with MS are significantly higher than in those without MS, whereas MPV is significantly lower. However, no such trend is observed in men [25]. This result suggests that platelet count and MPV might be a surrogate marker associated with clustered MS in women but not in men [26]. In a study conducted by Varol et al. [12], MPV was correlated with AHI and desaturation index in men, and MPV was correlated with AHI but not with desaturation index in women. The proportion of women was lower in our OSAS group, as compared to controls, although there was no difference in the OSAS subgroups. We performed a correlation analysis in subgroups of men and women but no correlation was found in the subgroup analysis between MPV and AHI in the OSAS group in men or women.

MPV is an indicator of platelet activation and also shows a close relationship with cardiovascular risk factors, such as diabetes mellitus, hypertension, hypercholesterolemia, obesity, and MS. A large population showed that the presence of MS and its components do not constitute a difference in MPV values in obese patients with a BMI ≥30 kg/m² [26]. Although we excluded patients with a diagnosis of diabetes, the severe OSAS group had a significantly larger waist circumference and lower HDL values, as compared to those in the control group.

Another reason for the lack of significance could be high variability in the automated MPV measurement method. A review indicated high variability in literature MPV values as a risk factor for cardiovascular disease; thus, a standardized method may be needed [27]. EDTA and citrate-based anticoagulated blood samples from the same patients were assessed with an autoanalyzer and there was a close correlation between MPV as measured by EDTA and citrate, but mean MPV measured from EDTA samples was 0.66 fL (9%) more than citrate. Those authors also stated that MPV can be measured accurately by both EDTA and citrate anticoagulation methods if the analysis is performed within 1 h of sampling [28]. The importance of timing was emphasized in a study designed to standardize MPV measurements. Optimal measurement time was 120 minutes after venipuncture. Platelet count was most stable in EDTA, and no inverse relationship is found between MPV and platelet count [29]. Therefore, standardized laboratory methods and time adjustments are essential when measuring MPV, and different measurement methods can cause different results.

The difference between our study and two other studies is exclusion of diabetes mellitus and ischemic heart disease as well as careful cardiac examinations. The limitations of our study are that we only measured MPV once for each patient and that this was a retrospective study, unlike the study done by Nena et al. [13], even though all blood samples were collected between 7 and 8 AM and analysed within 30 minutes in our hospital. We also used EDTA instead of sodium citrate in our study [30]. Also another limitation could be the use of automatic analyzers instead of manual microscopic counting [27].

## 5. Conclusion

Since the first description of platelets more than a century ago, more and more studies have focused on the association between platelet function and size in different diseases. Although studies have shown a correlation between severity of OSAS and MPV, MPV could not be correlated with severity of OSAS in nondiabetic nonischaemic patients. These findings question the suggestion that MPV might be a marker of OSAS severity, as recommended by earlier studies. Prospective data on platelet indices during the natural history of OSAS would be very useful to fully appreciate their prognostic significance in this disorder, as proposed in earlier studies.

## Figures and Tables

**Table 1:** Demographic and anthropometric characteristics of the patients.

	Group 1 (AHI < 5)	Group 2 (AHI 5–15)	Group 3 (AHI 15–30)	Group 4 (AHI > 30)	*P* value
Patient number	30	38	41	91	

Sex (male %)	15 (50%)	30 (78.9%)	33 (80.5%)	76 (83.5%)	0.002∗
Age (years)	38.43 ± 12.79	43.50 ± 12.15	47.27 ± 10.95	45.64 ± 10.15	0.006∗
Hypertension (%)	4 (13.3%)	8 (21.1%)	8 (19.5%)	23 (25.3%)	0.566
Smokers (%)	14 (46.7%)	18 (47.4%)	20 (48.8%)	62 (68.1%)	0.037∗
Pack-year smoking history	1.5 (10)	1.5 (17.25)	2.0 (15)	15 (24)	0.001∗
Patient number	30	38	41	91	
BMI (kg/m^2^)	26.91 ± 4.61	29.10 ± 4.51	30.01 ± 4.52	31.75 ± 4.53	0.001∗
Neck circumference (cm)	35.55 ± 3.63	38.45 ± 3.31	39.33 ± 3.31	40.71 ± 7.89	0.001
Waist circumference (cm)	92.17 ± 10.96	98.68 ± 8.76	101.61 ± 9.33	104.19 ± 14.26	0.001
Hip circumference (cm)	101.16 ± 7.35	103.74 ± 6.59	106.85 ± 16.54	107.98 ± 7.78	0.006
Waist to hip ratio	0.91 ± 0.075	0.95 ± 0.066	0.96 ± 0.099	0.97 ± 0.115	0.062

*P < 0.05.

**Table 2:** Sleep characteristics of the patients.

	Group 1 (AHI < 5)	Group 2 (AHI 5–15)	Group 3 (AHI 15–30)	Group 4 (AHI > 30)	*P* value
Patient number	30	38	41	91	

TST (min)	415.15 ± 50.81	383.33 ± 54.11	386.55 ± 57.39	380.59 ± 64.38	0.05
Sleep efficiency (%TST)	86.52 ± 9.09	80.47 ± 10.09	80.79 ± 10.03	83.03 ± 10.24	0.052
Stage 1 (%TST)	5.34 ± 1.92	6.72 ± 2.47	7.52 ± 2.87	10.6 (9.1)	0.001∗
Stage 2 (%TST)	51.98 ± 7.16	52.09 ± 6.53	50.83 ± 9.83	53.37 ± 10.26	0.510
Stage 3 (%TST)	20.86 ± 6.70	19.98 ± 7.19	21.00 ± 9.53	16 (13.2)	0.001∗
REM (%TST)	21.56 ± 6.32	20.72 ± 5.72	20.64 ± 6.57	17.08 ± 7.17	0.001∗
AHI (events/hour)	2.84 ± 1.41	9.58 ± 2.91	21.12 ± 3.88	54.17 ± 18.81	0.001∗
Average SpO_2_ (%)	96.79 ± 1.47	94.13 ± 12.65	95.35 ± 1.59	95.17 ± 1.54	0.296
Minimum SpO_2_ (%)	91.70 ± 3.88	88.55 ± 4.72	82.29 ± 12.82	74.98 ± 14.09	0.001∗
Time duration with SpO_2_ < %90	0.001 (0.001)	1.78 ± 4.04	3.5 (9.25)	23.1 (71.9)	0.001∗
ODI	1.5 (2.57)	7 (7.47)	20.85 ± 6.16	49.42 ± 20.41	0.001∗

TST: total sleep time; AHI: apnea hypopnea index; REM: rapid eye movement; AHI: apnea-hypopnea index; ODI: oxygen desaturation index; *P < 0.05.

**Table 3:** Full blood count and biochemical characteristics of the groups.

	Group 1 (AHI < 5)	Group 2 (AHI 5–15)	Group 3 (AHI 15–30)	Group 4 (AHI > 30)	*P* value
Patient number	30	38	41	91	

WBC (×10^3^ cell/*µ*l)	7.08 ± 1.37	7.27 ± 1.97	7.83 ± 1.79	7.89 ± 1.99	0.069
RBC (×10^6^ cell/*µ*l)	5.06 ± 0.58	5.23 ± 0.47	5.25 ± 0.48	5.19 ± 0.67	0.654
Hb (g/dl)	14.55 ± 1.42	14.97 ± 1.42	15.24 ± 1.40	15.08 ± 1.47	0.328
Htc (%)	43.76 ± 4.24	44.93 ± 3.89	44.29 ± 7.27	44.91 ± 5.87	0.808
PLT (×10^3^ cell/*µ*l)	271.57 ± 51.83	260.76 ± 64.52	266.76 ± 62.11	278.59 ± 84.64	0.597
MPV (fl)	9.21 ± 0.75	9.36 ± 0.94	9.33 ± 0.72	9.37 ± 1.02	0.910
PDW (fl)	15.71 ± 2.12	16.19 ± 2.34	15.67 ± 1.91	15.84 ± 2.88	0.810
Glucose (mg/dl)	94.34 ± 9.58	95.70 ± 11.35	100.91 ± 10.69	99.31 ± 14.30	0.079
Creatinine (mg/dl)	0.70 ± 0.15	0.81 ± 0.14	0.8 (0.19)	0.8 (0.2)	0.684
Total cholesterol (mg/dl)	201.92 ± 47.72	201.88 ± 42.05	210.01 ± 43.06	207.43 ± 40.21	0.758
HDL cholesterol (mg/dl)	54.03 ± 12.79	42.5 (11.5)	45.25 ± 12.72	40.48 ± 8.72	0.001∗
LDL cholesterol (mg/dl)	119.91 ± 41.91	119.62 ± 43.47	125.20 ± 41.56	127.49 ± 37.26	0.691
Triglyceride (mg/dl)	92.5 (83.83)	128.45 (113)	155.5 (155.6)	196.45 (113.7)	0.017∗

WBC: white blood cells; RBC: red blood cells; MPV: mean platelet volume; PDW: platelet distribution width; HDL: high-density lipoprotein cholesterol; LDL: low-density lipoprotein cholesterol; VLDL: very low density lipoprotein cholesterol; *P < 0.05.

Both HDL and triglyceride were found to be significantly high in severe OSAS group compared to normal population (*P*: 0.001 and  *P*: 0.013).

**Table 4:** Correlation analyses and *P* values of MPV and PDW with polysomnographic parameters in the whole group.

	Average SpO_2_ (%)	Minimum SpO_2_ (%)	Time duration with SpO_2_ < %90	Oxygen desaturation index	AHI (events/hour)
MPV	0.875	0.446	0.277	0.165	0.098
PDW	0.647	0.749	0.857	0.785	0.735

**Table 5:** Correlation analyses and *P* values of MPV and PDW with polysomnographical parameters in the severe OSAS group.

	Average SpO_2_ (%)	Minimum SpO_2_ (%)	Time duration with SpO_2_ < %90	Oxygen desaturation index	AHI (events/hour)
MPV	0.266	0.523	0.430	0.055	0.010∗
PDW	0.522	0.969	0.840	0.156	0.061

*P < 0.05.
